# Effect of Build-Up Strategy and Selective Laser Melting Process Parameters on Microstructure and Mechanical Properties of 316L Stainless Steel

**DOI:** 10.3390/ma19010026

**Published:** 2025-12-20

**Authors:** Krzysztof Żaba, Maciej Balcerzak, Paweł Pałka, Radek Čada, Tomasz Trzepieciński, Martyna Szczepańska

**Affiliations:** 1 Department of Metal Working and Physical Metallurgy of Non-Ferrous Metals, Faculty of Non-Ferrous Metals, AGH University of Krakow, al. Adama Mickiewicza 30, 30-059 Cracow, Poland; balcerzak@agh.edu.pl (M.B.); m.szczepanska779@gmail.com (M.S.); 2 Department of Materials Science and Engineering of Non-Ferrous Metals, Faculty of Non-Ferrous Metals, AGH University of Krakow, al. Adama Mickiewicza 30, 30-059 Cracow, Poland; pawel.palka@agh.edu.pl; 3 Faculty of Mechanical Engineering, VSB-Technical University of Ostrava, 17. Listopadu 2172/15, 708 00 Ostrava, Czech Republic; radek.cada@vsb.cz; 4 Department of Manufacturing Processes and Production Engineering, Faculty of Mechanical Engineering and Aeronautics, Rzeszów University of Technology, al. Powst. Warszawy 8, 35-029 Rzeszów, Poland; tomtrz@prz.edu.pl

**Keywords:** 3D printing, additive manufacturing, laser powder bed fusion, mechanical properties, microstructure, selective laser melting

## Abstract

Additive manufacturing, or 3D printing, is a method for creating three-dimensional objects layer-by-layer based on a digital model. This article presents the results of research on selective laser melting (SLM) of 316L stainless steel powder. Its aim is to investigate the relation between the mechanical properties of SLM-fabricated 316L steel samples obtained from uniaxial tensile tests and the SLM process parameters including the build-up strategy. Four different configurations of 3D printing orientation relative to the build platform were considered. The variable parameters of the SLM process were laser power and laser scanning speed. The morphology of the external surfaces and the microstructure of the SLM-processed samples were examined. The results show that samples printed in the longitudinal and transverse configurations had the highest tensile strength. Samples printed in the vertical and diagonal configurations had the greatest dispersion of values of mechanical parameters. The main difference in mechanical properties after doubling the SLM process parameters was a decrease in elongation for samples printed in the longitudinal configuration and an increase in this value for samples printed in the transverse configuration. The use of higher laser powers and laser scanning speeds guarantees a more compact, non-porous microstructure of SLM-processed samples.

## 1. Introduction

Additive manufacturing (AM) is a technology that uses the data contained in 3D geometric models to create objects with complex geometry made of metals or polymeric materials [1,2]. This is performed by successively depositing layers until the desired structure is achieved. Powder-based AM shortens the production time and reduces the waste of material, enables on-demand production, and allows the management of the target’s mechanical properties and the microstructure of the printed components [3]. AM-fabricated metal parts often have a finer microstructure than those produced traditionally, which can translate into increased strength [4,5]. Furthermore, AM enables the rapid repair of defects or damage in parts by precisely depositing powder in the desired locations [6].

Selective laser melting (SLM), along with selective laser sintering, multi-jet fusion, and electron beam melting are similar to powder bead fusion processes [7]. Laser powder bed fusion (LPBF) and SLM are two names for the same process of using a high-energy laser to fuse layers of metal powder to build 3D components. LPBF produces highly precise components, enabling dimensional control and the production of parts with internal channels [8,9]. The basic elements of an LPBF system include a closed chamber with a heat source (laser), a scanner, a powder bed, a powder feed arm for sintering subsequent layers, and usually a source of inert gas [10,11]. In LPBF, a high-intensity laser beam melts successive layers of metal powder. The parameters of this method, such as the scanning speed, layer thickness, and build-up direction, have a key impact on the quality of the manufactured components [12,13]. Shahriari et al. [14] emphasized that parts produced using SLM process are characterized by a microstructure completely different from their conventionally manufactured counterparts. These authors reported that cycles of high-temperature heating followed by rapid cooling contribute to the development of microstructural inhomogeneities related to dislocation density, grain size, and residual stresses. A number of these undesirable effects lead to anisotropic properties of SLM-produced components [15,16]. Appropriate parameter selection is crucial in SLM printing technology [17,18]. It depends on the type of material being processed and the geometric characteristics of the printed component. Important variables include the lengths of the scanned paths, powder feed rate, scanning speed, laser power, and laser spot size [19,20].

Investigations of the SLM process of 316L steel focus on how this process affects the material’s microstructure and defects as well as its mechanical, tribological, and corrosion properties, and how post-printing treatment can be optimized for a specific application. Ansari et al. [21] analyzed a multiphysics model that allows relating the process parameters to the temperature in the melt zone and, consequently, to the microstructure and defects. Thermal distortion of SLM-processed models made of 316L was the subject of Păcurar et al. [22], who demonstrated that precise parameter settings resulted in fewer pores and defects and improved mechanical properties of SLM-printed parts. In the same context, Wang et al. [23] demonstrated that cracks initiate at the pores and defects related to a lack of fusion, unlike for cast and rolled materials. Zhou et al. [24] described the submicron cellular structure of SLM-processed 316L steel components resulting from surface-tension-driven Benard conventions, reporting that elongated submicron cell structures always coexist inside one single macro-solidified grain. Li et al. [25] investigated the effects of the build-up direction and SLM process parameters on the tribological behavior of 316L steel. They found that the effect of the direction of the build-up on the wear rate is not significant. Mogyla et al. [26] focused on the effect of a post-processing heat treatment on the microstructure and mechanical parameters of SLM-processed 316L steel. The experimental tests showed that the elongation and tensile strength of SLM-printed samples did not meet the standards for cast and rolled 316L steel. Many studies indicate that SLM processes of 316L stainless steel use very different process parameters (laser power, layer size, scanning speed, scanning strategy, energy density) which makes it difficult to compare results between studies and draw general conclusions [27]. So far, few studies have paid attention to the combined effect of the SLM process parameters and different build-up directions on the static mechanical properties of 3D printed 316L steel. Therefore, the aim of this study is to investigate the relation between the parameters of the printing process and the direction of the build-up at constant energy density on the mechanical properties of SLM-processed 316L steel. Four different 3D printing configurations were considered. The external surface morphology and microstructure of the SLM-processed samples were also assessed. The results of the laboratory tests allow a better understanding of the relation between the process parameters and the final material properties in order to optimize industrial SLM technology.

## 2. Materials and Methods

### 2.1. Material

Selective laser melting was performed using 316L stainless steel powder according to the ASTM A240/A240M-22a [28] standard (1.4404 according to EN 10027-2 [29] a), supplied by Xact Metal (State College, PA, USA). The chemical composition of the 316L steel powder, according to the data supplied by the manufacturer [30], is presented in Table 1.

316L steel is an austenitic stainless steel that has been used in applications requiring resistance to acidic corrosive environments (organic and inorganic) in medicine, in the tool industry [31], and structural application [32]. This steel is characterized by good thermal conductivity, making it suitable for SLM processing, ensuring high dimensional accuracy of 3D printed components.

A globular metal powder intended for the XC200C metal 3D printer (Xact Metal, State College, PA, USA) was used to fabricate the samples. The distribution of the sizes of the particles of the powder, provided by the manufacturer [33] (determined according to the ASTM B822-20 standard [34]) is presented in Table 2. Selected mechanical parameters of the 316L steel determined based on the ISO 6892-1 [35] standard by the manufacturer of the 316L steel powder [31] are listed in Table 3.

### 2.2. Selective Laser Melting

**Figure 1:**
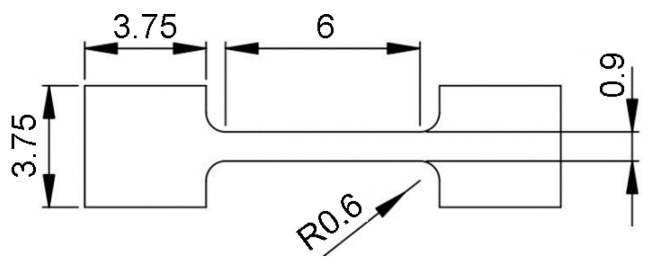
Technical drawing of the sample for static tensile testing with dimensions in mm.

**Figure 2:**
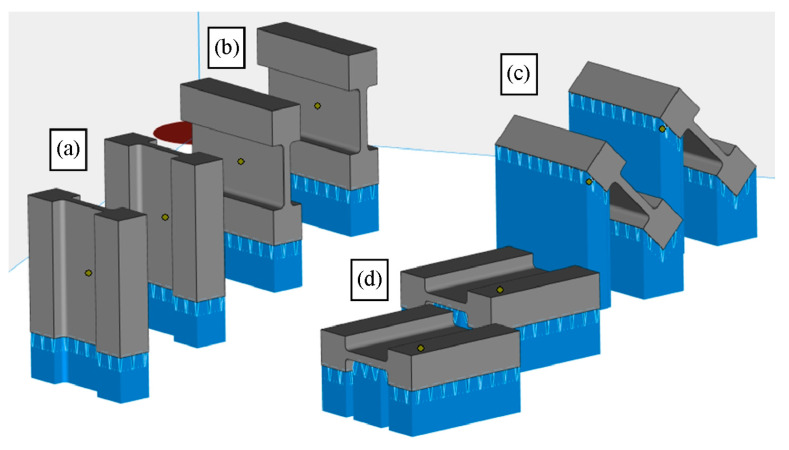
Visualization of the layout and print configuration profiles (gray—the actual print, blue—the supports): (**a**) longitudinal, (**b**) transverse, (**c**) diagonal, and (**d**) vertical.

The 3D printing process was performed using an XM200C (Xact Metal, State College, PA, USA) metal 3D printer, which uses a 200 W Yb fiber laser. Microsample models (Figure 1) for uniaxial tensile testing were designed in SolidWorks CAD program, version 2016 (Dassault Systèmes, Vélizy-Villacoublay, France). The CAD model was then converted using Materialise Magics (Materialise, Leuven, Belgium) software (version 25) to an .stl file with a triangular mesh applied. In this work, four configurations of SLM-processed samples were considered, differing in the spatial orientation of the model with respect to the printer’s build platform, the model’s build-up direction, and the laser scanning path (Figure 2). For the purposes of this work, the printing configurations are referred to as follows: longitudinal (Figure 2a), transverse (Figure 2b), oblique (a profile inclined at a 45° angle relative to the build platform) (Figure 2c), and vertical (Figure 2d). These configurations were designed to produce tensile samples so that the tensile direction of the resulting samples varied with respect to the model’s build-up direction and the scanning path.

**Figure 3:**
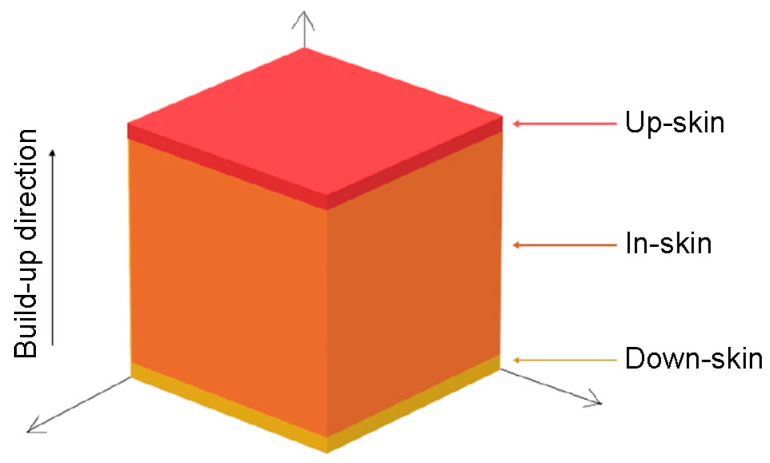
Schematic diagram of the 3D printed model divided into three areas depending on the build-up direction: down-skin, in-skin, and up-skin.

Figure 3 shows a schematic division of the 3D printed model into three key areas: up-skin, in-skin, and down-skin. Up-skin refers to upward-facing surfaces, down-skin refers to downward-facing surfaces (which often require support structures). In the tested samples, it can be assumed that the thickness of the up-skin and down-skin areas includes two layers of melted material. In-skin area refers to the core region between up-skin and down-skin areas [36,37]. Each layer of the model is created by melting selected areas of the powder layer, which consolidates and bonds to the layer below. In this work, the same SLM process parameters were used to build all layers of the SLM-processed structure.

In previous studies, this technological approach was not applied because the research focused solely on characterizing the material produced under standard processing parameters recommended by the machine manufacturer for 316L steel. In the present work, the selected parameter sets were chosen to maintain the same volumetric energy density while remaining within the stable operating window of the SLM system, enabling a controlled assessment of how altered thermal conditions influence the mechanical properties. The tests were conducted for varying laser powers and laser scanning speed (Table 4). The first set of SLM processing parameters used a 50 W laser at a scanning speed of 150 mm/s. In the second set, the laser power and scanning speed were 100 W and 300 mm/s, respectively. These parameters were selected to ensure the same energy density in both process parameter sets. The layer thickness during SLM of 316L powder, as recommended by the 3D printing machine manufacturer [33], was 30 μm.

**Figure 4:**
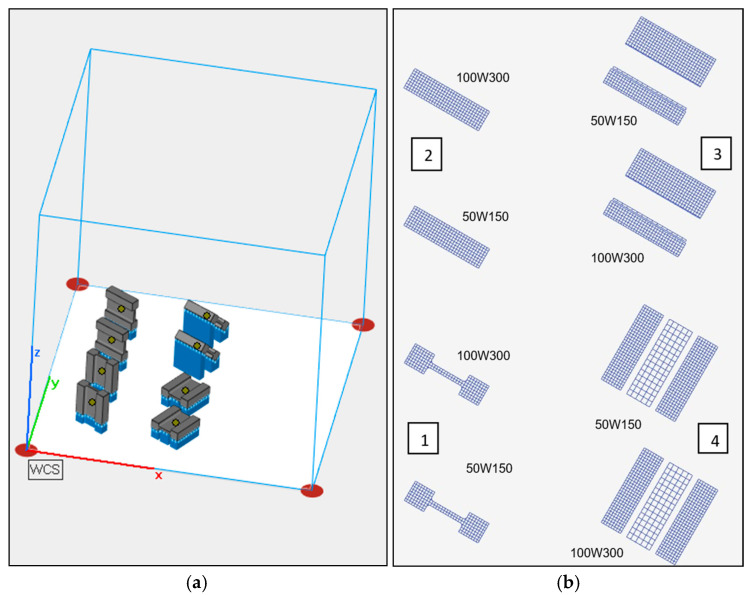
(**a**) Arrangement of samples on the build platform (gray—the actual print, blue—the supports) and (**b**) order of the laser operation.

The design phase considered the target configuration of the samples relative to the build-up direction and the arrangement of the supports (Figure 2). Their arrangement within the printer’s build platform was planned as shown in Figure 4a. Considering the change in SLM process parameters, two different combinations of laser power and laser scanning speed were considered (Figure 4b). The samples were marked with xWy symbols, where x denotes the laser power and y denotes the laser scanning speed. The laser operated alternately, moving in the order of areas 1 to 4, as shown in Figure 4b.

### 2.3. Uniaxial Tensile Test

**Figure 5:**
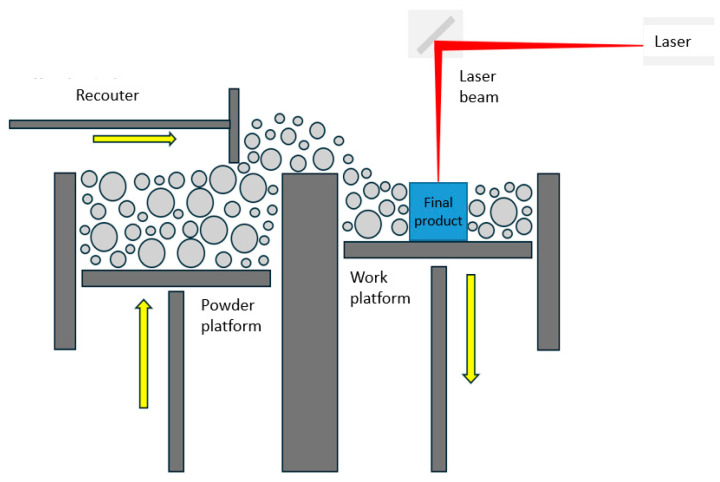
Sample production scheme (arrows indicate the direction of movement).

**Figure 6:**
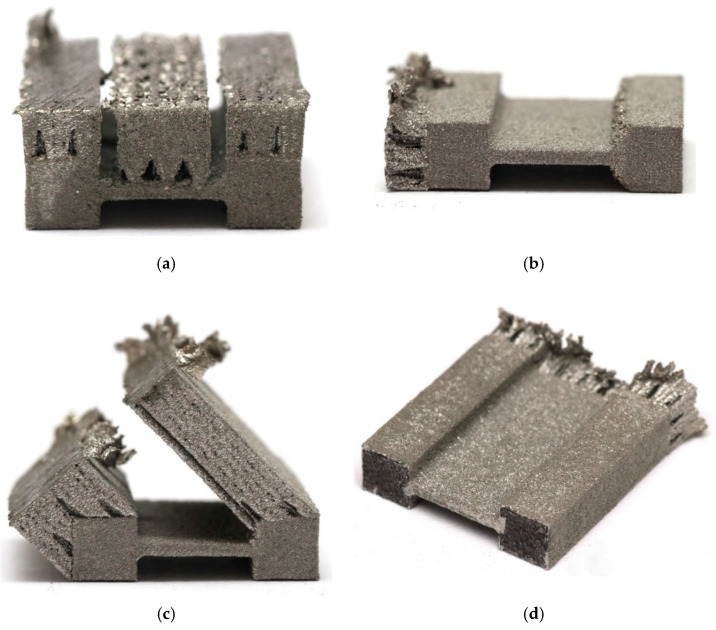
Photographs of SLM-processed samples (50W150) at different orientations: (**a**) vertical, (**b**) transverse, (**c**) oblique, and (**d**) longitudinal.

**Figure 7:**
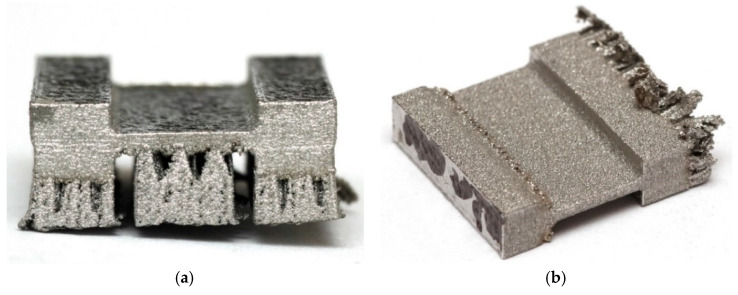

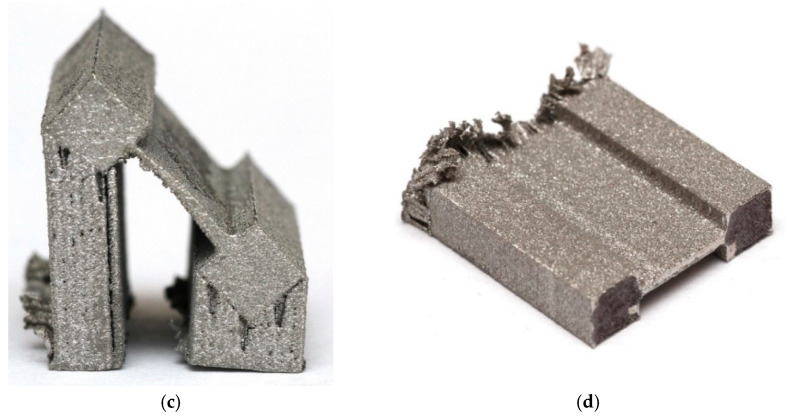
Photographs of SLM-processed samples (100W300) at different orientations: (**a**) vertical, (**b**) transverse, (**c**) oblique, and (**d**) longitudinal.

**Figure 8:**
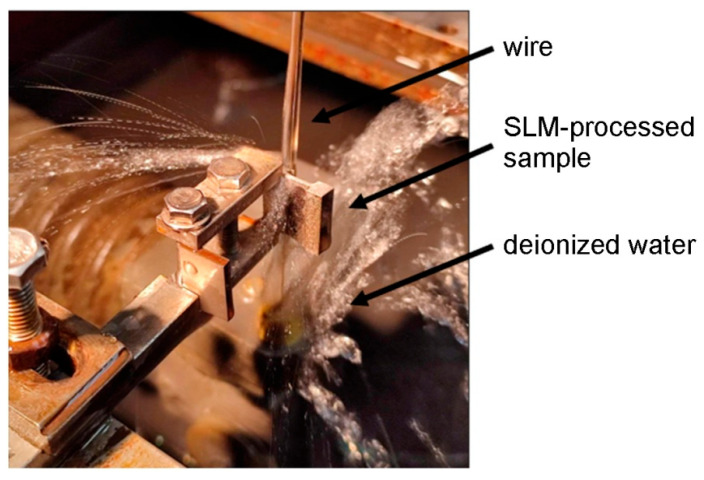
Photograph of the process of cutting off print supports using a wire EDM machine.

The basic mechanical properties of the SLM-processed 316L steel samples were determined by a uniaxial tensile test. The sample production scheme is presented in Figure 5. The first preparatory step for the uniaxial tensile test was to cut off the supports in the 3D printed samples (Figure 6 and Figure 7). The supports were first cut from the surface of the SLM machine’s build platform and then from the surface of the finished sample. For this purpose, a wire electrical discharge machining (EDM) machine (Figure 8) was used. The use of an EDM machine was aimed at minimizing surface defects that could affect the results of the uniaxial tensile test.

Static tensile tests were performed using a TM–SM tensile testing machine (Instron Corporation, Canton, MA, USA) according to the ISO 6892-1 [35] standard. The samples were stretched at a constant rate of 10^−3^ s^−1^ at ambient temperature. To ensure the repeatability of the results, three repetitions were performed for each configuration, and the average value of the mechanical parameters was determined, including the yield stress R_p0.2_ which is a measure of the stress at which a material experiences a permanent plastic deformation of 0.2%, the tensile strength R_m_, and the elongation A_10_.

### 2.4. Analysis of the Microstructure

**Figure 9:**
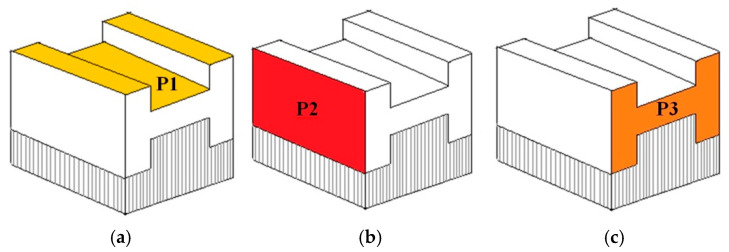
Surfaces of the vertical sample subjected to SEM: (**a**) P1, (**b**) P2, and (**c**) P3.

The analysis of the microstructure was divided into two stages. The first stage involved observing the microstructure of the two samples that were SLM-processed in a vertical configuration. This was to compare the microstructure of SLM-processed samples using lower (50 W and 150 mm/s) and higher (100 W and 300 mm/s) 3D printing process parameters. Three surfaces of each sample were analyzed (Figure 9). The second stage of microscopic examination involved observing the microstructure of the 100W300 sample printed in a vertical configuration.

Microstructural analyses were performed using a ZEISS optical microscope (Carl Zeiss AG, Oberkochen, Germany) and a scanning electron microscope (SEM) S3400N (Hitachi, Tokyo, Japan). The samples were ground and polished using a Labo Force 3 metallographic polisher (Struers, Bellerup, Denmark). Abrasive papers with grits ranging from 600 µm to 4000 µm were used. Subsequently, polishing was performed using a 1 µm diamond paste. Finally, the samples were polished using a silicon oxide suspension and etched using Kroll (New York, NY, USA) and a modified Nital reagent.

## 3. Results and Discussion

### 3.1. Tensile Test

**Figure 10:**
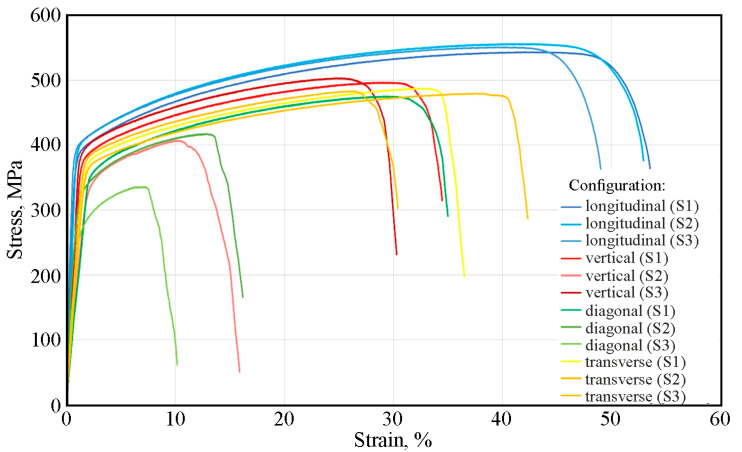
Stress–strain curves after static tensile test for SLM-produced samples at laser power of 50 W and laser scanning speed of 150 mm/s.

Figure 10 shows the stress–strain curves for the four tested printing configurations using a laser power of 50 W and a scanning speed of 150 mm/s. The samples printed in the longitudinal configuration are characterized by high repeatability of results. The average yield strength was 379 MPa, tensile strength 550 MPa, and elongation 51%. In contrast to the longitudinal configuration, the samples printed in the vertical configuration exhibit greater dispersion in results. The stress–strain curve for sample S2 deviates from the others, showing a significantly lower tensile strength—nearly 100 MPa lower—and elongation nearly half that of the S1 and S3 samples. This is likely due to the fact that the samples subjected to stretching were cut from three areas of the printed sample: the in-skin core, the up-skin, and the down-skin. As with the vertical configuration, diagonal-printed samples were characterized by significant dispersion in the obtained stress–strain curves. Figure 6c shows the uneven thickness of the reduced section of SLM-printed sample, and the flexure of this section is also visible. This is likely due to the printing configuration and the resulting thermal stresses, which caused a deformation of the printed sample. The samples printed in the transverse configuration, similarly to the samples printed in longitudinal configuration, are characterized by very stable values of the yield strength of approximately 349 MPa and tensile strength of approximately 483 MPa (Figure 10).

**Figure 11:**
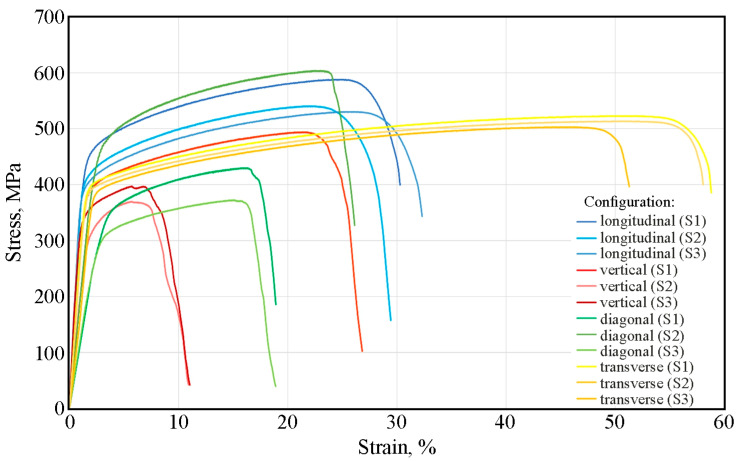
Stress–strain curves after static tensile test for SLM-produced samples at laser power of 100 W and laser scanning speed of 300 mm/s.

**Figure 12:**
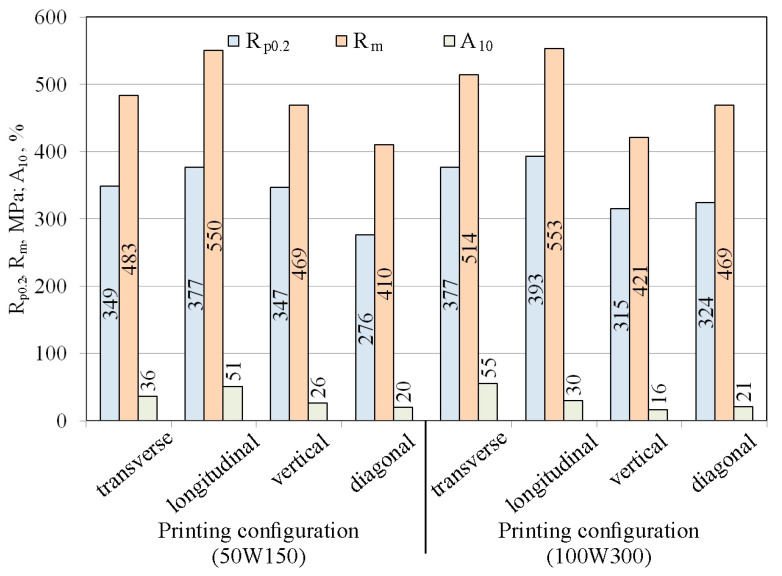
Values of R_p0.2_, R_m_, and A_10_ for the SLM-fabricated samples from the static tensile test.

Doubling the laser power and scanning speed within the same print configuration changed the mechanical properties (Figure 10). For samples printed in the longitudinal configuration, the yield strength and tensile strength varied within the range of 369–408 MPa and 531–588 MPa, respectively. The average elongation was 30% (Figure 11 and Figure 12). Interestingly, compared to lower print parameters (Figure 10), both R_p0.2_ and R_m_ reached similar values. However, the difference is visible in the percentage of elongation, which decreases from 50% for the 50W150 samples to 30% for the 100W300 samples (Figure 12).

The stress–strain curve of the second stretched sample (S2) printed in a vertical configuration differs in its tensile strength and elongation from the other curves for samples printed in the same configuration. The values of these parameters for S1 and S3 were higher than for S2 and more similar to the average parameters of the samples printed in a diagonal configuration (Figure 12). For S2, the elongation value A_10_ = 27% was more than twice as high as the elongation of the other SLM-printed samples in a vertical configuration. In the group of samples printed with a 100 W laser and a laser scanning speed of 300 mm/s, the samples printed in the vertical configuration showed the lowest values of the analyzed mechanical parameters (Figure 12).

The large dispersion in the values of the mechanical parameter of the samples printed in the diagonal configuration (Figure 11) may be related to the origin of the samples from different areas, including down-skin, in-skin, and up-skin. Furthermore, the sample flexure (Figure 6c) induced by residual stresses could also have influenced the results. The samples printed in the transverse configuration showed a small dispersion in their values of yield strength and tensile strength (Figure 11).

For reference, the tensile properties of cold-rolled 316L stainless steel, as reported by Lackey [38], include yield strength YS = 357 MPa, ultimate tensile strength UTS = 629 MPa, and Young’s modulus E = 212 GPa. Sazgar et al. [39] reported the following mechanical properties of as-rolled 316L material: YS = 468 MPa, UTS = 688 MPa, and elongation A = 30.9%. Hot-rolled 316L stainless steel has the following properties [40]: YS = 263 MPa, UTS = 589 MPa, and Young’s modulus E = 174 GPa. Aziz et al. [27] conducted an extensive literature review indicating that 316L steel in as-cast condition exhibits the following mechanical properties: YS = 200–250 MPa, UTS = 450–550 MPa, and A = 30–35%. Wrought 316L stainless steel is characterized by YS = 170 MPa, UTS = 480 MPa, and A = 40% [27]. The yield strength values of SLM-fabricated samples presented in this paper were higher than the yield strength values of wrought [27], hot-rolled [40], and as-casted [27] 316L material. The UTS values for samples manufactured with various printing configurations were lower than those of 316L steel in as-hot-rolled condition. SLM-fabricated samples in transverse and longitudinal configurations showed UTS values within the range of as-cast samples [27]. Only samples printed in the transverse configuration showed greater elongation compared to as-rolled [38] and as-casted [40] 316L steel. Most of the analyzed printing configurations provided lower elongation values compared to wrought 316L stainless steel [27]. Young’s modulus values were similar (E = 198 GPa) than those presented in the literature for hot forged (E = 198 GPa) [41], cold rolled (E = 210 GPa) [42], and wrought material (E = 189 GPa) [43].

### 3.2. Analysis of Surface Morphology and Microstructure

#### 3.2.1. Morphology of External Surface

The microstructure of the samples printed in the vertical configuration was observed using a scanning electron microscope. Calignano et al. [44] noted that factors influencing the surface texture of 3D-printed components include, among others, powder particle size distribution, thermal conductivity, layer thickness, and the post-processing method. Three surfaces (Figure 9) were observed to analyze track overlapping, the level of metal powder melting in the up-skin area, and to assess the surface quality of the samples in the as-printed condition.

**Figure 13:**
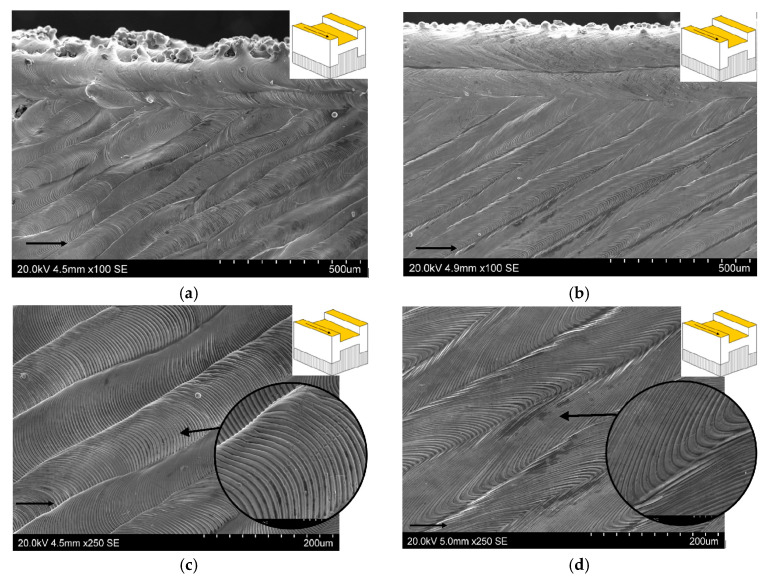
SEM micrographs of the side edge of samples: (**a**) 50W150 and (**b**) 100W300. Morphology of the scan tracks and ripple formations on samples: (**c**) 50W150 and (**d**) 100W300 observed in the P1 (yellow) surface (arrows in SEM micrographs and on the yellow surface indicate the printing direction).

Figure 13a,d present a comparative analysis of P1’s surface quality (Figure 9a) for two parameter groups: 50W150 and 100W300. SEM micrographs show that this surface is characterized by a texture free of pore defects. Laser tracks and their overlaps are visible, consistent with the applied laser rotation angle (67°). The purpose of rotating the laser by a value greater than 45° was to achieve a non-overlapping effect between fused layers and to prevent undesirable anisotropy of the properties of the sample printed by SLM.

Figure 13a,b illustrate the differences in the shape of the tracks left by the displacement of the weld pool on the sample surface. Lower laser power and scanning speed ensured a uniform shape of these tracks; however, these SLM process parameter values affect the surface morphology of the successively melted layers. Doubling the laser scanning speed causes curving of the fish scale patterns, resulting in greater uniformity and compactness of the individual layers.

The side edges of the samples (Figure 13a,b) show embedded particles of powder that have not completely melted. Lower scanning speed and laser power (50W150) results in poorer print surface quality (Figure 13a) compared to samples built with higher SLM process parameters (Figure 13b). Lower laser power and scanning speed prevent the powder from fully melting, resulting in a rougher texture.

Figure 13c,d show enlarged views of the sample surface, illustrating the melting zone’s morphology. During the building of the 50W150 samples (Figure 13c), fish scale patterns are distributed symmetrically with respect to the direction of the movement of the laser beam. Meanwhile, the SLM process with higher laser power and scanning speed reveals uneven material solidification and uneven fish scale patterns on the face along the tool path (Figure 13d). A fish-scale weld bead (also called a rippled bead) forms due to the periodic solidification and movement of the melted pool of material during the SLM process.

**Figure 14:**
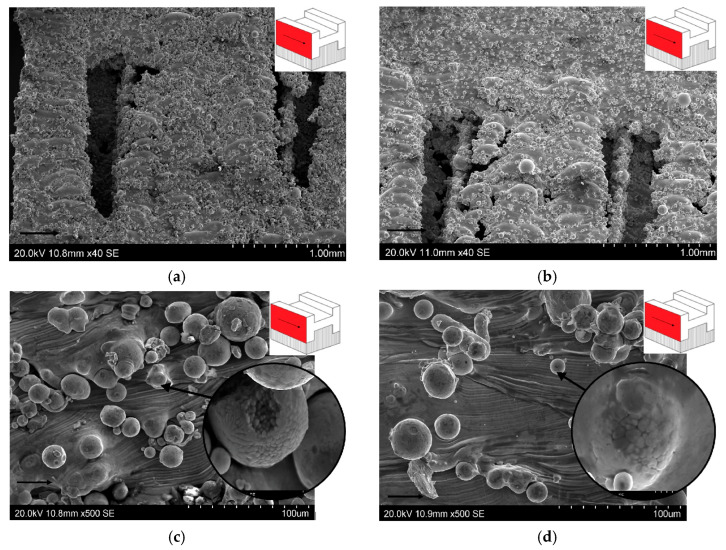
SEM micrographs of supports on the surface of P2 of samples: (**a**) 50W150 and (**b**) 100W300. Morphology of scan tracks on the surface of P2 (red) of samples: (**c**) 50W150 and (**d**) 100W300 (arrows in SEM micrographs and on the red surface indicate the printing direction).

Figure 14a,d present SEM micrographs of the surface of P2 (Figure 9b). The visible voids in Figure 14a,b are the spaces between supports. Within the two analyzed parameter groups (50W150 and 100W300), there is a noticeable difference in the accumulation and size of spheroidal metal powder embedded in the sample surface. The sample fabricated at laser power 50 W and scanning speed 150 mm/s (Figure 14a,c) is characterized by a higher density of metal powder present on the surface.

Figure 14c,d show the central area of the sidewall of the SLM-processed sample using a vertical configuration. Melted layers of intersecting laser tracks were observed—similar to that observed on the surface P1 (Figure 13). On the surface of P2 of the 100W300 samples, the observed area also shows a denser surface area filled with fused but incompletely melted spheroidal powder (Figure 14c), compared to the 50W150 sample (Figure 14d). The high temperature of the melting process and the subsequent rapid cooling led to secondary crystallization of the molten powder [45]. The thermal effect of the subsequent fused layers affects the processes of recrystallization of the previous layers of the material [46]. The enlargements in Figure 14c,d show images of a crystallized metallic droplet. The direction of the growth of the grains in Figure 14c is consistent with the direction of heat removal. The resulting crystallites within the depth of fusion have different shapes. Equiaxed and columnar crystallites can be distinguished (Figure 14c,d).

**Figure 15:**
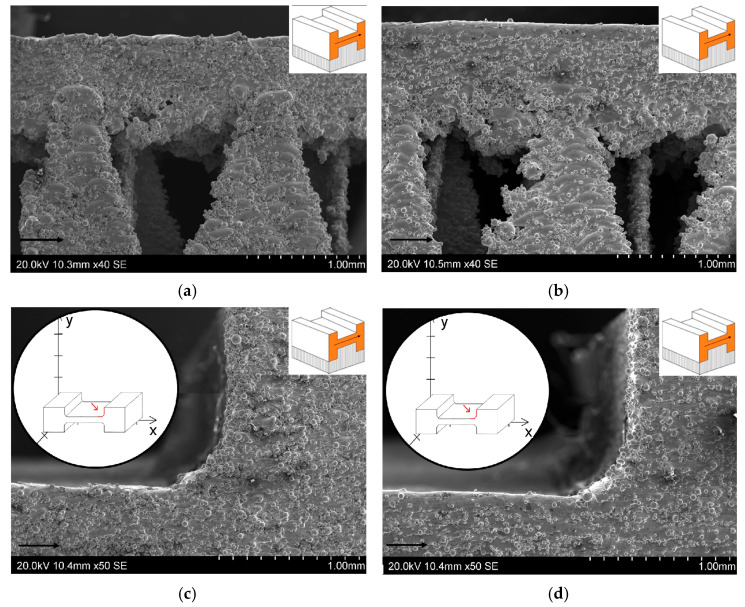
SEM micrographs of supports on the surface of P3 of samples: (**a**) 50W150 and (**b**) 100W300. A view of the rounding zone of samples on the surface (orange) of P3 of samples: (**c**) 50W150 and (**d**) 100W300 (arrows in SEM micrographs and on the orange surface indicate the printing direction).

The last surface observed was the second sidewall (P3 in Figure 9c), which included the support area (Figure 15a,b) and the arc area of the reduced section of static tensile test specimen (Figure 15c,d). The upper portion of the SEM micrographs, shown in Figure 15a,b, confirms that the upper surface area of the specimen (up-skin area) is characterized by high quality and smoothness. A clear difference is visible between the surface quality of the 50W150 sample (Figure 15a) and the 100W300 sample (Figure 15b). Higher laser power and scanning speed ensure better surface smoothness. Additionally, less metal powder solidifies in the last layers (up-skin area), which reduces the roughness of the surface. It is worth noting that even naked-eye observation of the sample after cleaning it with alcohol revealed that the surface of the 50W150 sample is more matte than the surface of the 100W300 sample. This indicates lower light reflection by the sample surface, and therefore poorer surface smoothness. Additionally, it should be noted that there is an irregular surface at the rounded profile of the samples (Figure 15c,d). It can be concluded that this affects the mechanical properties of the samples fabricated in this configuration, as evidenced by the stress–strain curves in Figure 10. These curves indicate that the gravitational collapse of unsupported layers, as well as the difficulty in precisely separating the supports from this surface, results in a large dispersion in the values of the obtained mechanical parameters. This is also confirmed by the observations of Calignano et al. [44], who noted that gravity influences the melting of unsupported layers, which collapse into the unmelted powder beneath the currently melted layer. This results in a significantly rougher surface on the bottom side of the component (down-skin area) than on the surfaces of up-skin area. This disparity between these two surfaces is exacerbated by the non-uniform rate of heat diffusion between the powder and the solid. This creates temperature gradients that disrupt the equilibrium in the molten metal region and contribute to the irregular shape of the individual layer edges. Additionally, Figure 15a,b show voids on the uneven bottom surface of samples. These voids can initiate future cracks in the printed structure that affect the static tensile test results.

Figure 15c,d show a fragment of the lateral surface of the samples, taking into account the profile arc. Observation of the samples showed that higher SLM parameters positively influence the more accurate reproduction of the arc rounding. The slower laser scanning speed and associated longer cooling time cause the previously melted layer to solidify before the next layer is applied. Consequently, the subsequent powder layers do not fully fuse together, and a “stepped” arc effect is more likely to occur. The fusion of metal powder particles and adjacent layers through laser beam melting influences the final mechanical properties of components manufactured using the SLM method [47]. One of the key problems and challenges associated with SLM is the tendency for residual stresses to develop during the melting of the metal powder and the joining of subsequent metal layers [48,49]. Depending on the intensity of these stresses and whether they relax, cracks in the material and undesirable changes in the shape of the printed structure are possible [50]. Residual stresses arise as a result of rapid, intense heating and subsequent cooling [51,52].

#### 3.2.2. Microstructure

**Figure 16:**
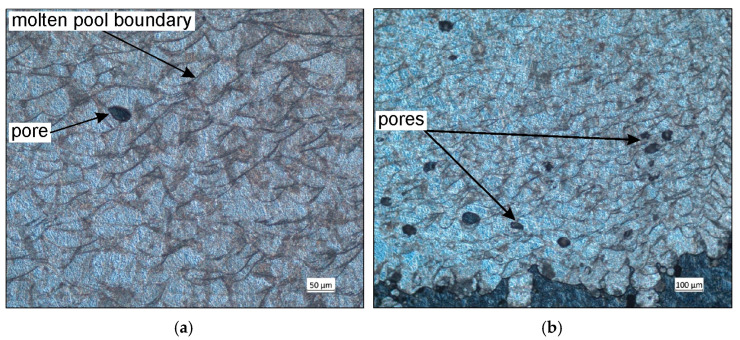
Optical microscope images of (**a**) in-skin and (**b**) down-skin areas of samples SLM-printed in vertical configuration.

The microstructure of a cross-section of a sample SLM-processed in a vertical configuration was examined. Clear boundaries were observed between individual layers of solidified metal. The dark traces visible in Figure 16a,b are arcs of the layers melted by the laser. The traces are not uniformly distributed, which is due to the rotation of the laser by 67° with each powder layer deposited. The cross-section also contains unconnected, randomly distributed pores (Figure 16a,b). The pore density increases the closer they are to the up-skin area. Considering that 316L steel powder dedicated to the Xact Metal printer was used for printing, it can be assumed that the risk of “gas pores” is low. Therefore, the character of the SLM process should be considered the cause of the porosity. In this work, the same printing parameters used in the down-skin, in-skin, and up-skin areas resulted in residual stresses in the SLM-printed samples. Figure 16b shows a fragment of supports located in the down-skin area of the sample, which shrank and deflected upward. The highest pore density is observed in the same area. Combining microstructural observations with naked-eye observations suggests that the samples printed in the vertical configuration are characterized by the highest porosity, which in turn results in reduced mechanical properties.

**Figure 17:**
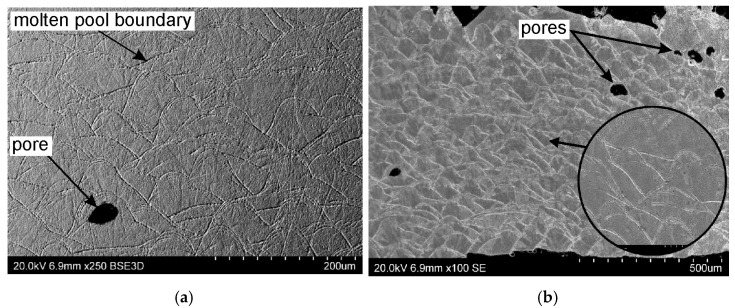
SEM micrographs of down-skin areas of samples SLM-printed in vertical configuration at different magnifications: (**a**) ×250 and (**b**) ×100.

Observations of the microstructure in a cross-section of the sample fabricated in vertical configuration were also made using a scanning electron microscope. These observations are consistent with those from an optical microscope. A characteristic feature of the SLM process is the overlapping or interconnected scanning traces, visible in Figure 17a,b. SEM micrographs of the cross-section reveal a characteristic pattern formed by semicircular lines, which are the melt boundaries of individual traces. Their shape is the result of melt pool solidification. The overlapping of the molten layers ensures the best possible material continuity of the sample [53,54]. However, the tested sample contains discontinuities in the form of closed pores of similar size. The risk associated with porosity in the SLM-processed sample primarily involves the potential formation of cracks and a shorter lifespan. This is therefore a valuable observation from the perspective of the potential use of printed 316L steel parts on an industrial scale; combining a laser scanning speed of 300 mm/s with laser power 100 W is not optimal for components that require, among other things, high strength.

## 4. Conclusions

The orientation of the printed sample relative to the build platform, the laser power, and the scanning speed influence the mechanical properties of the finished samples. The results of the static tensile tests showed that the best mechanical properties, across both laser power and scanning speed parameters, were achieved by samples printed in the longitudinal and transverse configurations. The greatest dispersion of mechanical parameters was observed for samples printed in the vertical and diagonal configurations. The main difference in the mechanical properties of SLM-printed samples after doubling the laser power and scanning speed is a decrease in elongation for samples printed in the longitudinal configuration and an increase in elongation for samples printed in transversal configuration. The greater scatter of mechanical parameters in vertical and diagonal samples can be explained by the orientation of interlayer boundaries relative to the loading direction. These orientations result in more interfacial regions that are prone to defects, such as porosity or incomplete fusion. Even when the same energy density is applied, differences in heat flow and layer consolidation lead to uneven melting and cooling, affecting microstructure uniformity and residual stresses. Consequently, the structural features and mechanical variability are directly linked to the energy expended during the SLM process.

According to the manufacturer of the 316L steel powder, the tensile strength of samples printed in the longitudinal configurations should reach 607 MPa. The tensile strength obtained during testing longitudinally printed samples at a laser scanning speed of 150 mm/s and a laser power of 50 W was 550 MPa. Similarly, the tensile strength (553 MPa) for samples printed in the longitudinal configuration at a laser power of 100 W and a laser scanning speed of 300 mm/min is lower than that declared by the powder’s manufacturer. Similarly, experimental tests resulted in yield strengths lower than those declared by the manufacturer.

The results of microscopic observations correspond to these conclusions regarding the mechanical properties. Selecting the appropriate printing parameters allows managing the product’s microstructure, which in turn determines the mechanical properties of the printed samples. The morphology of the fused layers, the presence of unmelted metal powder and pores, and the surface roughness of SLM-printed samples are parameters that change with variations in the scanning speed and laser power. Using higher scanning speeds and laser powers can positively impact the surface quality of SLM-printed samples. Using higher values of these parameters guarantees a more compact structure of SLM-processed material. This involves depositing less metal powder on the surface, which in turn ensures greater surface smoothness of the sample’s sidewalls.

## Figures and Tables

**Table 1:** Chemical composition (wt.%) of 316L stainless steel.

Cr	Ni	Mn	Mo	Si	O	N	P	S	C	Fe
16.00–18.00	10.00–14.00	≤2.00	2.00–3.00	≤1.00	≤0.10	≤0.10	≤0.04	≤0.03	≤0.03	balance

**Table 2:** Size distribution.

Characteristic Diameter of Particles, μm	Minimum Fraction, vol.%	Maximum Fraction, vol.%
d90	40	60
d50	25	35
d10	15	25
<16	N/A	5

**Table 3:** Mechanical parameters of 316L steel (as-manufactured condition).

Parameter	Value
Tensile strength in the horizontal (XY) direction, MPa	607
Tensile strength in the vertical (Z) direction, MPa	572
Yield strength in the horizontal (XY) direction, MPa	455
Yield strength in the vertical (Z) direction, MPa	455
Elongation in the horizontal (XY) direction, %	37
Elongation in the vertical (Z) direction, %	44
Young Modulus, GPa	192

**Table 4:** SLM printing parameters used.

Parameter	Set 1	Set 2
Laser power, W	50	100
Laser scanning speed, mm/s	150	300
Laser diameter, mm	0.10	
Scanning angle, °	67.0	
Stripe size, mm	20.0	
Layer thickness, mm	0.030	
Hatch distance, mm	0.024	

## Data Availability

The original contributions presented in this study are included in the article. Further inquiries can be directed to the corresponding author.
